# Hospital and Institutional News

**Published:** 1918-09-28

**Authors:** 


### HOSPITAL AND INSTITUTIONAL NEWS.

### THE SHERBURN HOSPITAL FOUNDATION.

The Sherburn Hospital at Durham, whose embarrassing plethora of funds we noticed last week, has had a long and honourable career. It was founded in the latter half of the twelfth century by Hugh Pudsey, Bishop of Durham, who collected in it all the lepers in his diocese, and made a handsome endowment for their maintenance. It was one of several hundreds of leper-houses or of hospitals originally founded for the maintenance of lepers, that was investigated by the Commissioners of Henry VIII. after the great dissolution of the monasteries; for all leper-houses and other hospitals, including almshouses, were originally ecclesiastical establishments, and, though not actually monastic, had a strong monastic flavour and a semi-monastic constitution. Most of them imitated the monasteries not only in their government and regimen, but also in their abuses. As leprosy died out, and no lepers remained to claim their hospitality, their funds were appropriated to the enjoyment of the master or warden, and of the chaplains, clerks, canons, or other ecclesiastics to whom the government of the hospitals and the administration of their funds had been entrusted by pious founders. All such hospitals were ruthlessly suppressed, dissolved, and abolished by the Commissioners of Cromwell; but those that, like Shireburn or Sherburn Hospital at Durham, St. Leonard's Hospital at York, St. John the Baptist at Canterbury, St. Bartholomew's Hospital in London, and a few others, who devoted their funds, in the absence of lepers, to the poor and the sick, were suffered to remain and to retain their possessions, which, being chiefly in land, have greatly enhanced in value in the seven or eight hundred years since they were founded. Thus it is that good Bishop Pudsey's hospital still flourishes and enjoys an income of £24,000. Had all the hospitals of ancient foundation been as honest and as thrifty, there would be little need in these days for Hospital Saturdays and Hospital Sundays, or for incessant appeals for funds. So true is it that :—

The evil that men do lives after them :
The good is oft interred with their bones.

### HOSPITAL NEEDED FOR MALTA.

The sinister work of the U-boat in Mediterranean waters is brought home to us by Lord Methuen, the Governor of Malta, when he states, in a letter to the press, that the "Merchant Sailors' Rest," a branch of the British and Foreign Sailors' Society, has received during the war over 10,000 men, women, and children of all nationalities, rescued from torpedoed ships. Excellent though the work of the institution is, there is urgent need in Malta, in the opinion of the Governor and of others best qualified to judge, of a hospital under British management. The scheme has been enthusiastically taken up and a site has been found, close to the Grand Harbour, the estimated cost of which is £7,000. Captain G. A. L. Wisely and Mrs. Wisely have generously offered to give a sum of money for an endowment equivalent to any sum raised for building a hospital. Lord Methuen hopes that before he leaves the island he will have laid the foundation-stone of a hospital in every respect a worthy memorial of his comrades who have given their lives for the Empire.

### THE PATTERAN IN FRANCE.

The American Red Cross is providing the American Army in France with thousands of white cloths marked with a big red cross at one end and a red arrow at the other. These indications will be distributed by Red Cross bearers and runners in the wake of the advancing troops, so that the wounded who are left behind and able to walk may be guided to the nearest first-aid station. By means of these marks much suffering should be saved, and perhaps even the lives of men who would otherwise lose precious time in having their wounds attended to. Of course, the plan is only a simple adaptation of the gipsy trail known to readers of the "Romany Rye," "Aylwin," and "Katerfelto" as the patteran, or, more correctly, "patrin." This was a handful of leaves or twigs placed at the cross-roads by gipsies to indicate to later comrades of the road the direction taken. The points were set along the intended way.

Save by the Old Road none attains the new,
And from the Ancient Hills alone we catch the view.

### A SECRETARY'S RECOLLECTIONS.

Mr. Walter Davies, secretary to the Royal Infirmary, Preston, has taken advantage of a recent occasion to recall the changes which have taken place at the infirmary during the past twenty-one years. His own experience of the hospital is somewhat longer. It was, he said, the Diamond Jubilee scheme of 1897 which began the transformation of the hospital on modern lines. With a modest fund of £7,000 then raised an ambitious scheme costing three times as much was suggested. In the end £40,000 was raised and spent without a bazaar or sale, but simply by a flood of circulars. The district received £12,000, and met with a fine response. This circular is now historic, and we trust that the text has been preserved. The result was, continued Mr. Davies, that when Sir Henry Burdett paid a surprise visit there years ago, "we were, I believe, the only hospital in the country which received a clean bill of health. There was nothing in our management, construction, or equipment with which he had fault to find, and he gave us an excellent report." Since then, Mr. Davies is no less proud to state, the hospital is free from debt, except in regard to maintenance, and, if the maintenance account is overdrawn by £2,016, on the actual working the deficit is £16 only.

### SHELL-SHOCK CASES AND UNEMPLOYMENT.

The results of the Country Host Institution for the treatment of shell-shock cases have been sufficiently successful to lead the Ministry of Pensions to extend its scope to pensioners in all areas of the United Kingdom. A few months ago the Director-General of the Medical Department of the Navy approved the scheme for the benefit of undischarged naval ratings who were suffering from shell-shock. A beginning has been made with an after-care scheme, in the sense that an attempt is made to find suitable work for each patient at the end of his treatment. It is said that most of those who have finished the course are now usefully employed. We devoutly hope that this is no exaggeration. Unfortunately, however, the extent of unemployment from which even undischarged officers are suffering is far more serious than the general public has any idea. The agencies, official and unofficial, are crowded with applications with which they have been unable to deal. An unofficial agency of old standing confessed the other day that it had the names of 500 discharged officers upon its books for whom no suitable posts as yet had been found; and that the number was likely to increase. We have no reason to believe that its experience was exceptional.

### A FINE COTTAGE HOSPITAL CHAIRMAN.

The death of Sir Richard Glyn has robbed the Victoria Cottage Hospital, Wimborne, of a generous benefactor. When the hospital was established in 1887 in commemoration of Queen Victoria's Jubilee, Sir Richard Glyn became chairman of the trustees, and the institution was formally opened by his wife. Sir Richard continued chairman for twenty-seven years, until he resigned in 1914 because of his great age. His benefactions included a new east wing, which was built and equipped at his expense in 1892. In his will Sir Richard left £100 free of legacy duty to the hospital. Mr. George H. Solly has succeeded the late baronet as chairman.

### TWO MAYORS TO THE RESCUE.

The Mayors of Wandsworth and Battersea have had to assist the governors of the Bolingbroke Hospital at Wandsworth Common. The strain of four years of war have been severe, and has resulted in an overdraft of £8,000 at the bank. The deficiency is accounted for by the increase in the price of provisions and commodities. The cost of administration showed no increase. The Mayors suggest that a subscription of sixpence per head of the population of the two boroughs would produce £12,500, and this would clear the deficit and leave a substantial balance for working expenses. In addition to the appeal of the two Mayors for subscriptions, they ask the churches and chapels to make special collections on behalf of the hospital. A local Flag Day has resulted in a reasonable amount being raised, and we hope that the tide has turned at last.

### TRAMWAY STAGES AND OUT-PATIENTS.

Now that tram fares and the distance between each stopping-place are liable to modification (since when fares are not raised stages are apt to be shortened), the interests of out-patients should be remembered by hospital committees whose patients used to make use of a tram that stopped by the hospital's doors. An alteration in such case places out-patients in a dilemma. Either he or she has to pay an extra penny to reach the hospital or else an extra walk of, say, seventy yeards each way has to be endured. Either plan may be a considerable hardship, for pennies are often scarce and out-patients should not be compelled to add an avoidable walk to the appalling tedium of waiting which a visit to an out-patient department almost invariably means. Since the Insurance Act has made matters worse instead of better, and the numbers of out-patients, the effect of the war apart, are otherwise maintained, the hospital again can come to the rescue. It can easily approach the tram authority, and after pointing out the number of patients affected, can make a strong case for letting the old stage, whatever it was, end at the hospital's gate.

### MEDICAL MEN ON PUBLIC BODIES.

At least two members of the Hove Corporation seem to forget that a healthy population costs the rates less than an unhealthy one. One of these two members of this Corporation, who is a nonagenarian, by the way, quibbled the other day about paying the Baby Week deficit of £21. Fortunately, his military duties permitted a medical member of the Corporation to be present at the meeting, and his rejoinder quickly put an end to all criticism, and the two members found themselves a very small minority. The incident emphasises the value of medical men on our governing authorities, and in matters Imperial the doctor M.P. is no less useful. Baby Week has come to stay, even in Hove, and the work of the Maternity and Child Welfare Committee, once undertaken, must be maintained.

### PRIVATES DAVID AND JONATHAN.

Two striking coincidences have recently occurred at the Boscombe Hospital. The first instance concerns two young men belonging to Sussex, who joined the Army at Brighton at the beginning of the war. After being sent to various training camps they were in due course sent out with the early drafts of Kitchener's Army to France. The affection of these two was in some ways stronger than is often to be found even amongst brothers, and it was a great delight to them that in the war they were not divided. Both were in the same regiment, both were in the same company, and both were in the same platoon. They saw a great deal of fighting in the front trenches, and both were wounded within two days of one another. One of them was judged sufficiently ill to be returned to "Blighty," and at Dover he heard incidentally that he was to be forwarded to Boscombe Hospital.

There he was received by the resident medical officer, whose duty it was to allot the different cases to the different wards, and the bearers were directed to hand him over to the sister of the King George Ward. The other man's case was more obscure, and after a time he went back to the trenches, was again wounded, and this time sent home. He found himself at length at Dover, where he entrained for an unknown destination. In due course the train arrived at Boscombe, and he was admitted by the officer-in-charge to the King George Ward, where he settled down comfortably. After a few days he was allowed "up," and on going to the ward kitchen the first person he met was his friend. The second case is even more striking, and concerns two brothers who joined the Australian Forces early in the war. These men enlisted on the same day, their regimental numbers being consecutive. They trained together, and served for thirty months in France without parting. They were both gassed on the same day, came to England on the same hospital ship, and arrived at the hospital together, where they found themselves side by side in the same ward. After a short stay these men were discharged to a convalescent depôt, still together.

### THE ISSUE OF CERTIFICATES IN POOR-LAW INFIRMARIES.

It has been common in Poor-Law infirmaries to grant to panel patients certificates weekly as to their condition and the nature of their illness. This has been done at Lambeth Infirmary until the early part of last year, when the practice was discontinued, on the ground that there was no legal obligation for the Guardians or their medical superintendent to grant them. The matter was placed before the National Health Insurance Committee, which communicated with the Local Government Board. The latter have now informed the Lambeth Guardians that in its opinion inmates of Poor-Law institutions should be furnished on their request with certificates of the nature and duration of their sickness. This opinion has not satisfied the Guardians, who, before reverting to their old procedure, are pressing the Board for the legal authority compelling them to issue the certificates. Whilst this legal squabble is going on between the Local Government Board and the Guardians, the patients in the infirmary are compelled to suffer through not getting the certificates entitling them to the benefits under the Act. It is hardly too much to say that anything which can delay or annul benefits to the unfortunate insured person is supported by official machinery, and anything which can make the benefits real or useful is obstructed. No wonder the Act is hated by its "beneficiaries," the sick poor.

### LAMBETH GUARDIANS EXPLAIN.

Lambeth Board of Guardians has received a report from the medical officer of the infirmary with reference to the man whose leg was amputated and who was discharged without crutches, as mentioned in the last issue of The Hospital. The doctor states that the man's leg was amputated for gangrene of the foot, and at the time of his discharge from the infirmary he had quite recovered from the operation. His discharge was given to him at his own request, and his wife offered to provide a taxicab to take him home; the question of an ambulance had not been raised. They were of opinion that the wife was able to provide crutches for him, as neither he nor she made application to take crutches out with them. It was, of course, their custom to give patients crutches to take out with them if they needed them. Evidently there was an oversight on both sides. The wife failed to ask for the crutches, and the officers did not ask her if she required them. This apparently is the only explanation that can be offered for sending the man out of the infirmary without them, and probably the publicity given to this slight oversight may lead to the simple question being asked by the officers in future. With regard to the money retained by the officers, they had no right to return it on their own initiative. The man was a Southwark patient, and had a message been sent by telephone to the offices of the Southwark Guardians permission to refund the man the money would have been granted readily. This is virtually a confession of error, and we hope it will prevent any repetition of the kind.

### SCHOOL DOCTORS AND PICTURE PALACES.

Most medical officers recognise how the picture palace may spread infection, and Dr. Millard, the medical officer of health for Leicester, refers to the arrangements for children's performances in Leicester, whereby a charge of one penny only is made and the entertainment lasts for two hours on Saturday afternoons. They are greatly patronised, and as many as 1,000 or 1,600 children are often crowded together. Therefore the Sanitary Committee, acting on a suggestion made to Dr. Millard by Dr. Warner, the School Medical Officer, has approached the Watch Committee asking if the children's performances cannot be duplicated, and the performance itself and the number of children allowed to be present at a time cannot be reduced by one-half. As one hour's entertainment for one penny is still extremely good value it is unlikely that the total attendance would be much curtailed. If a short interval between the performances were insisted upon, the building between times could be thoroughly aired.

### THE OPPOSITION TO WELFARE CENTRES.

Dr. Harry Shore, medical officer of health for Walsall, implies in his report that some doctors have not been keen supporters of the Infant Welfare Centres established by the municipality. Exception was taken to medical treatment being given, and many of the cases received were said to have been unsuitable for the centres, which should have referred them to their own medical attendants. Dr. Shore's reply was that the local faculty had contended that they were too busy to devote the requisite time and attention which the public demanded. Some of the mothers concurred in the view, and the belief was that the Centres would relieve this strain. Dr. Shore points out that this type of criticism tends to curtail the usefulness of the work. Not infrequently mothers have refused to take children to their own doctor when requested to do so, generally alleging inability to pay. Hence those at the Welfare Centres had to choose between turning the cases away or receiving them.

### THE WANT OF DRESSINGS IN HOLLAND.

The want of wound-dressing requisites in Holland has become for the moment extremely acute. The price of cotton wool and gauze is ever on the advance, and other absorbent materials are as yet not in use. With sphagnum moss, which grows abundantly in the Low Countries, the Dutch general practitioner is absolutely unacquainted, whilst the importation of wood-pulp cellulose has been stopped and the stocks of this valuable material are exhausted. The reclaiming of soiled dressings is the order of the day now. Several manufacturers are trying an experiment with thread of peat (turf) to make it fit for use as a substitute for absorbent cotton and gauze. The results are for the moment satisfactory.

### AN OPPORTUNITY FOR THE RICH DRUGGISTS.

A strong appeal is being made by the Pharmaceutical Society for subscriptions from pharmacists and others engaged in the drug trade to a fund known as "The War Auxiliary Benevolent Fund," which has just been started. The Council is confident that pharmacists and their assistants who have been "denied the high honour of making the personal offering of substance and service, which has cost so many of their *confrères* unspeakable anguish of mind and bodily torture, and fearful if glorious death, while those who have remained in safety and comparative comfort and ease will rejoice in the opportunity of sharing the burden, by cheerfully and generously giving of their means to resettle and re-endow in civil life the men who have resigned so much for them." Here is an appeal impossible to resist, and pharmacists are expected to give not an odd five-pound note. but twice as much as they can afford. It is common rumour that some of the wholesale druggists have, during the war, amassed great wealth; if this be true, there is little doubt that they will give of their abundance, for they have always been staunch supporters of pharmaceutical benevolent funds.

### ANOTHER GERMAN RUSE FRUSTRATED.

It is common knowledge that before the war the Germans practically monopolised the synthetic drug industry. Such commonly prescribed drugs as phenacetin, antipyrine, and aspirin were German products; aspirin, which is as familiar to the present generation as salts and senna were to our grandfathers, was the property of the German firm of Bayer and Co., whose works at Leverkusen, near Cologne, are said to be one of the principal depôts for the manufacture of poison gas. Aspirin, antipyrine, phenacetin, and the rest are now being manufactured not only in this country, but in France, Spain, the United States, Japan, and elsewhere, so that after the war Germany will find that she has not only lost the monopoly, but is face to face with powerful competitors in a most lucrative trade that goes hand in hand with the dyestuffs industry, the monopoly in which she has also lost. But if we think that the German manufacturers are sitting still and doing nothing under this rebuff we sadly deceive ourselves. Their cunning minds are at work—but they are not cunning enough. The New York police have arrested two directors and four other members of the American branch of the Bayer Company; they are charged with conspiring to retain rights in alien property after the said property had been taken over by the Public Custodian. It is alleged that they had secured control of another company, which they were using merely as a cover for the interests of the Bayer Company, their object being to keep alive the German dyestuffs and drug industries and to secure a basis on which to reorganise the industry as soon as the war is over. It is to be hoped that the proper authorities in this country are keeping as sharp a lookout for this sort of ruse as the Americans are.

### THE SPANISH EPIDEMIC IN HOLLAND.

Holland, too, has been lately vexed by an attack of the so-called "Spanish grippe," which is nothing else, in the opinion of the Dutch medical men, than a common influenza epidemic. This epidemic was very acute and infectious, but it had in general a benign character. Only a few deaths are reported through secondary pulmonary affections. As the stocks of the most generally used antipyretic medicines in Holland are nearly exhausted, the average medical practitioner has confined himself to administering only a julapium.

### THIS WEEK'S DRUG MARKET.

Most of the price changes are again in an upward direction, and the improved tone of business is maintained. Another considerable rise in the value of Japanese refined camphor has taken place. Menthol is quieter, but the advanced price is fairly well maintained. Pancreatin and pepsin are dearer and only little of either is available. Japanese dementholised peppermint oil, citric acid, dill seed, sassafras oil, and star aniseed oil are also quoted at higher prices. The upward movement in the value of aspirin continues, but phenacetin still has a slightly downward tendency. The scarcity of benzoic acid and sodium benzoate is more acute. There is practically no change in quinine and bromides.

### TO OUR READERS.

**Contributions are specially invited from any of our readers to these columns. They should deal with topical subjects and news. They must be authenticated for the information of the Editor only. The minimum payment if published is 5s. There is no hard-and-fast rule as to space, but notes of about twenty lines in length are preferred.**

